# A Novel NPHP5 Gene Mutation in Three Siblings With Nephronophthisis Without Retinitis Pigmentosa: A Case Report

**DOI:** 10.1155/crig/1453255

**Published:** 2025-04-28

**Authors:** Randah Abdullah Dahlan, Roaa Hani Fairoozy

**Affiliations:** ^1^Department of Internal Medicine, King Abdullah Medical City, Mecca, Makkah Province, Saudi Arabia; ^2^Department of Laboratory, King Abdullah Medical City, Mecca, Makkah Province, Saudi Arabia

**Keywords:** nephronophthisis, nephronophthisis-related ciliopathies, NPHP, NPHP5, renal ciliopathies

## Abstract

Nephronophthisis (NPHP) is a hereditary renal disorder characterized by the progression to end-stage renal disease (ESRD) at a young age. Our understanding of this disorder continues to improve as we identify more genes and gene variants associated with NPHP. In this report, we present a young patient with newly diagnosed advanced renal impairment and a strong family history of ESRD at a young age. The patient's kidney biopsy showed features suggestive of severe chronic interstitial nephritis, along with histopathological findings of advanced renal disease. Genetic testing revealed a novel variant in the *IQCB1*/NPHP5 gene, which is autosomal recessive. Family genetic analysis revealed that the patient's parents and two of his children are heterozygous for the identified variant, while two siblings with ESRD are homozygous for the *IQCB1* p.(Ala486Asp) variant. Unlike previously described mutations in the *IQCB1*/NPHP5 gene, the patient and his affected siblings do not have retinitis pigmentosa. We report this novel gene variant in a Saudi family, describe its associated clinical features, and present the results of the family segregation analysis.

## 1. Introduction

Cilia are membrane-bound organelles that protrude apically from the surface of nearly all mammalian cell types [1]. These organelles play important roles in tissue development and function [1]. For example, cilia on the surface of the renal tubular epithelium are important for maintaining tubular structure, and any disruption leads to cyst formation. Likewise, cilia of the photoreceptor cells of the retina convert light into signals, which are then sent to the brain via the visual pathway, and any defects result in retinal diseases [2]. Mutations in genes encoding for ciliary proteins result in a group of diseases known as ciliopathies. These ciliopathies often affect the kidneys as well as other organs like the retina, cerebellum, and liver [1]. Renal ciliopathies include autosomal dominant polycystic kidney disease (ADPKD), autosomal recessive PKD (ARPKD), and nephronophthisis-related ciliopathies (NPHP-RC) [1, 3]. NPHP-RC can present as isolated NPHP, NPHP with extrarenal features that do not constitute a recognizable syndrome, or syndromic NPHP (e.g., Bardet-Biedl syndrome, Joubert syndrome, and Senior-Loken syndrome) [1, 3]. Although most patients have isolated NPHP, some patients may manifest some syndromic features over time [3]. Clinical variants of NPHP are associated with specific defects in genes or specific gene variants. For example, the NPHP5 variant is associated with a mutation in the IQ calmodulin-binding motif–containing protein 1 (IQCB1) gene. This gene, named for the amino acids isoleucine (I) and glutamine (Q), is typically linked to progressive retinitis pigmentosa in combination with NPHP [4]. While visual impairment usually becomes apparent within the first year of life, kidney cysts and ESRD tend to develop later, typically during late adolescence [5]. Neurological symptoms such as developmental delay and seizures are rare, and hepatic involvement is not commonly observed [5].

In this report, we describe a patient with a novel mutation in the *IQCB1*/NPHP5 gene and discuss the associated clinical features and the results of family segregation analysis. Although it is recognized that all patients with variants of the NPHP5 gene have retinitis pigmentosa [3], this reported patient and his affected siblings do not have retinitis pigmentosa.

## 2. Case Report

A 32-year-old man, the index case, was referred from a peripheral hospital in the south of Saudi Arabia to our tertiary medical city in Makkah, Saudi Arabia, for a kidney biopsy. He was found to have impaired renal function tests during routine laboratory testing. At the time of presentation to our center, he had no active complaints. He has no known chronic medical illnesses, and no previous surgeries, apart from a history of intraocular lens implantation (IOL) for both eyes 10 years ago for amblyopia. His parents are first-degree cousins and have no chronic medical illnesses. He has four brothers and three sisters, with a younger brother who is 32 years old and has been on chronic hemodialysis for almost 4 years. This brother was diagnosed with ESRD in his late 20 s, and a report of a kidney biopsy performed in another hospital indicated the diagnosis of advanced IgA nephropathy with moderate interstitial inflammation. It is worth noting that this brother has a history of amblyopia in both eyes since childhood, and a report of his last ophthalmological examination done in 2020 showed that he was able to count fingers only but had no findings to suggest retinitis pigmentosa. He has no neurological or hepatic manifestations. The patient also has a sister who is currently 42 years old and was diagnosed in her late 20 s with advanced chronic kidney disease of unknown cause. She did not have a kidney biopsy performed at that time and received preemptive living renal transplant from her husband in the same year of diagnosis. There was no report of any ophthalmological involvement affecting the sister, nor does she have neurological or hepatic manifestations. The remaining patients' siblings have no chronic medical diseases. The patient, the index case, has recently started on sodium bicarbonate, alfacalcidol and folic acid tablets. He is a physician, nonsmoker, married and has four children.

His physical examination was unremarkable. An official ophthalmological assessment revealed that he has a vision of 6/7 in the right eye and 6/12 in the left eye. His pupils were round and reactive, and the cornea of both eyes was clear. He had bilateral phakic intraocular lenses, a myopic fundus with mild astigmatic disc in both eyes, normal retinal vasculature, and no signs of retinitis pigmentosa.

His initial investigations are listed in Table 1. A kidney biopsy was subsequently performed, and its microscopic examination revealed diffuse global glomerulosclerosis, severe interstitial fibrosis and tubular atrophy, and severe chronic interstitial nephritis with the inflammatory infiltrate consisting mainly of lymphocytes and eosinophils. Immunofluorescence testing showed segmental minimal glomerular fine granular staining for IgA (1+) and Kappa (1+) and Lambda (1+) light chains with no staining for IgG, IgM, C3, C1q, and fibrinogen. Electron microscopy examination showed no immune-type dense deposits.

The possibility of autosomal dominant tubulointerstitial kidney disease (ADTKD) was considered, and therefore, molecular genetic analysis for ADTKD was requested. The test was done at BioScientia International Laboratory Center in Frankfurt, Germany, using next-generation sequencing (NGS). The panel included six genes: *DNAJB11, HNF1B, MUC1, REN, SEC61A1,* and *UMOD*. However, the data showed no causative mutation identified in ADTKD-related genes. For further investigations, Whole exome sequencing (WES) was subsequently performed. A variant in the *IQCB1* gene, autosomal recessive (OMIM accession number 609254), was identified, where both alleles of the index case are affected by the variant c.1457C > A; p.(Ala486Asp). The identified missense variant at position 486 in exon 14 led to an amino acid change from Alanine to Aspartic Acid. Although the *IQCB1* gene is linked to NPHP and renal diseases, the identified variant was not described in the literature; thus, it was classified as a class III variant i.e., a variant of uncertain significance (VUS).  Table 2 shows the reported result.

**Figure 1:**
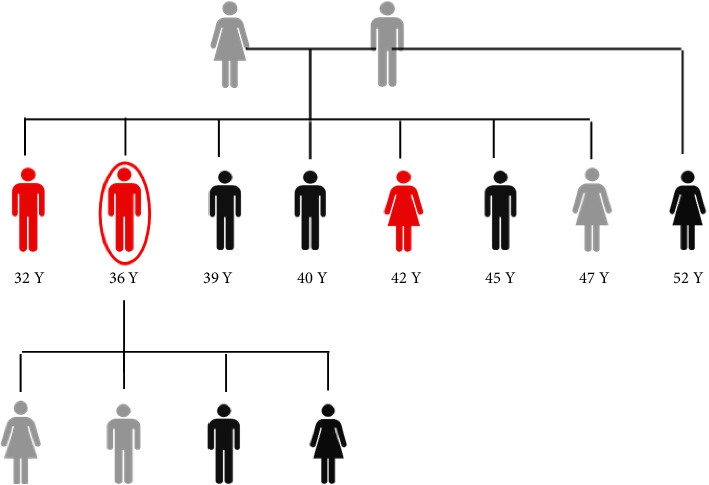
Pedigree of the patient's family. Circled symbol represents the index case. Symbols in red represent symptomatic/affected siblings, symbols in grey represents asymptomatic family members tested heterozygous for the identified variant, symbols in black represent untested family members. Numbers listed below symbols represent age in years. 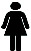: female gender, 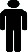: male gender.

**Figure 2:**
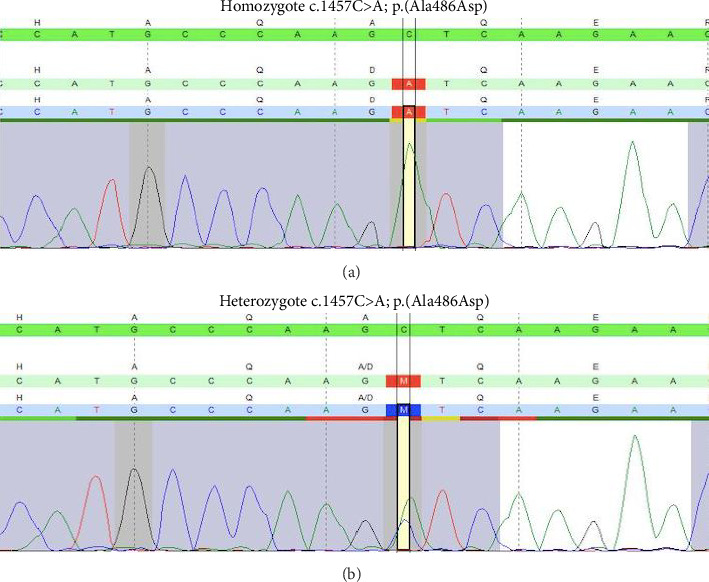
Sanger sequencing analysis of exon 14 of the *IQCB1* gene performed in the patient and his tested family members. The electropherogram shows a base change, distinguishing homozygous (a) from heterozygous (b) patterns associated with *IQCB1*, c.1457C > A; p.(Ala486Asp) mutations.

To prove the hereditary nature of the variant, family segregation analysis at three family levels was performed, including the index case's parents, three of his siblings, and two of his children. Blood samples were sent to CENTOGENE Laboratory in Germany to perform targeted Sanger sequencing for the relevant *IQCB1* regions. The targeted genomic position is evaluated (Reference sequence for *IQCB1*: NM_001023570.2). The genetic analysis showed that both parents are heterozygous for the *IQCB1* p.(Ala486Asp) variant. The variant heterozygosity was also found in one of his sisters, who is 47 years old and not known to have chronic renal disease (unaffected), and two of the index case's children. The data also showed that the affected siblings described above are homozygous for the same variant for the *IQCB1* p.(Ala486Asp). The family pedigree and the results of their mutation analysis are shown in Figure 1. The results of Sagner sequencing analysis are shown in Figure 2.

Bioinformatic in silico programs were used to predict a pathogenic effect for this variant. It was reported that Alanine 486 is located in a highly conserved domain, and 6 out of 8 in silico tools predicted a deleterious impact for this variant.

## 3. Discussion

NPHP is a kidney disease that is inherited in an autosomal recessive manner [1]. It is caused by a mutation in one of the genes that encode proteins involved in the function of primary cilia, basal bodies, and centrosomes [1, 3, 6]. More than 26 different genes have been identified as being involved in the development of NPHP, with mutations in the NPHP1 gene being the most common [6]. The clinical presentation of NPHP can vary depending on the specific gene involved, including the age of onset and the presence of extra-renal manifestations [3]. Common renal manifestations include impaired urinary concentrating ability and sodium reabsorption, bland urinalysis, and chronic tubulointerstitial nephritis which can progress to ESRD at an early age [3, 6]. Of note, these renal manifestations are non-specific, and may mimic the presentation of other renal disorders like ADTKD. Extra-renal manifestations could include the eyes, cerebellum, liver, and bones. Eye involvement may manifest as ptosis, myopia, coloboma of the chorioidea, optic nerve atrophy, nystagmus, strabismus, hyperopia, amblyopia, and isolated oculomotor apraxia [2, 7]. Importantly, patients with NPHP may also have a concomitant retinal involvement, ranging from dysplasia to degeneration, also referred to as retinitis pigmentosa, tapetoretinal degeneration, or retinal-renal dysplasia [7]. The concomitant occurrence of NPHP with retinitis pigmentosa represents Senior-Løken syndrome [7]. Retinitis pigmentosa occurs in approximately 10% of patients with NPHP types 1 to 4, without a clear genotype-phenotype correlation [7]. However, it has been observed that patients with mutations in the NPHP5 gene consistently exhibit retinitis pigmentosa [3, 7]. The NPHP5-gene, also known as *IQCB1* gene, is located on chromosome 3q21 and encodes nephrocystin-5, which interacts with calmodulin protein and the retinitis pigmentosa GTPase regulator (RPGR) protein [3, 6]. These proteins (i.e., nephrocystin-5, RPGR, and calmodulin) are localized to the connecting cilia of photoreceptors and the primary cilia of renal epithelial cells [3]. This specific localization or co-expression may explain the retinal phenotype in NPHP type 5. In NPHP5, retinitis pigmentosa typically presents early in life with blindness occurring by the end of the third year [3, 7]. However, it is important to note that the three patients described in this report did not have retinitis pigmentosa. This challenges the previous understanding that retinitis pigmentosa is always present in NPHP type 5 [3, 7]. A previous study has attempted to explain the variation in phenotype among patients with similar disease alleles, and one of the proposed explanations was that some of the transcripts of the affected alleles may escape a regulatory mechanism and transcribed into a partially functional protein [8]. Another proposed explanation was the effect of the genetic background, that is, variations in other genes which may alter the pathogenicity of a disease-causing allele [8]. Even without these explanations, it is known that identical mutations within the same gene can result in different clinical findings, due to the concept of mutation penetrance in genetic diseases [9]. With regard to the amblyopia observed in the index case and one of his affected siblings, it is worth noting that amblyopia has been previously reported in associated with certain types of NPHP [2, 7]. However, to date, there have been no documented cases linking amblyopia specifically to NPHP5.

The histopathological diagnosis of IgA nephropathy in the brother of the index case described in this report is noteworthy. The index case patient also exhibited minimal glomerular fine granular staining for IgA on immunofluorescence staining of the kidney biopsy. A previous study of Korean pediatric patients with NPHP13 reported similar findings, where two patients in one family had IgA deposits on their kidney biopsies, along with severe tubulointerstitial changes [10]. In that study, familial IgA nephropathy in these two patients was excluded based on the result of genetic tests [10]. Another patient from a different family in the same study had multiple immune complex deposits (IgG, IgM, C3, and C1q) on their kidney biopsy [10]. Another reported case of an adult patient with NPHP12 showed some IgA deposits with mild mesangial proliferation and chronic tubulointerstitial nephritis on kidney biopsy, but IgA nephropathy was clinically excluded due to the absence of hematuria or proteinuria [11]. It is important to note that IgA nephropathy is an inflammatory glomerulonephritis that can occur in association with systemic diseases or other glomerular diseases [12, 13]. IgA deposits can also be observed on kidney biopsies of individuals without evidence of kidney disease [14]. Therefore, while IgA nephropathy may contribute to tubulointerstitial inflammation and the progression of renal disease in patients with NPHP, its presence should be interpreted cautiously as it may be coincidental rather than causal.

The management of NPHP patients with renal involvement primarily involves supportive care, similar to managing any chronic kidney disease patient. However, knowing the underlying disease is crucial for providing prognosis, early detection of asymptomatic affected siblings, appropriate evaluation of potential kidney donors within the family, and offering premarital counseling to patients and their siblings, particularly in cases of consanguineous marriage. This approach was applied to the index case, who received supportive management for his chronic kidney disease, along with counseling regarding the nature of his condition and its prognosis, including the anticipated progression to ESRD. Given that the disease follows an autosomal recessive inheritance pattern and both parents are heterozygous carriers of the pathogenic variant, the untested siblings have a 25% chance of being affected, a 50% chance of being asymptomatic carriers, and a 25% chance of being completely unaffected and not carriers. Accordingly, it was strongly recommended that this risk be discussed with their family physician. His heterozygous sibling and children are expected to remain asymptomatic and are not at risk of developing clinical manifestations of the disease. Furthermore, they may be considered potential kidney donors, if necessary.

The molecular basis of NPHP5 has been previously studied to gain insights into mitigating NPHP5 deficiency, and in vitro modeling studies suggest that gene therapy could be a promising future therapeutic approach for patients with *IQCB1*/NPHP5 [15, 16]. However, drugs that target negative regulators of the ciliogenic pathway in cells depleted of NPHP5 are currently not available.

In summary, this report describes a novel variant of the *IQCB1* gene (NPHP5) in three siblings with significant renal involvement. To the best of our knowledge, this variant has not been reported previously. Unlike previously described patients with NPHP type 5, these three patients did not exhibit retinitis pigmentosa. Clinicians should be aware of the diagnostic challenge posed by the presence of IgA deposits in patients with NPHP. In addition to describing the clinical features associated with this novel variant, this report highlights the increased risk of rare autosomal recessive disorders in offspring from consanguineous marriage. It is important to monitor siblings of NPHP patients for the development of renal disease, especially those presenting with unexplained eye manifestations at an early age, to enable early detection and follow-up.

## Figures and Tables

**Table 1:** Index case investigations. Patients' laboratory investigations.

Test item	Reference range	Result
Hemoglobin (mmol/L)	13.5–17.5	11.3
Platelet (× 10^9^/L)	150–400	381
White blood cells (× 10^9^/L)	3.9–11	6.2
Sodium (mmol/L)	136–145	139
Potassium (mmol/L)	3.5–5.1	4.2
Bicarbonate (mmol/L)	22–29	23.9
Blood urea nitrogen (mmol/L)	2.86–8.57	10.7
Creatinine (mmol/L)	65.4–119.34	339.57
Corrected calcium (mmol/L)	2.12–2.52	2.02
Phosphorus (mmol/L)	0.81–1.45	1.65
Parathyroid hormone (ng/L)	15–68.3	713
Uric acid (mmol/L)	208.1–428.2	410.4
Hemoglobin A1C (%)	< 5.8	5.1
Albumin (g/L)	35–52	47
Total bilirubin (μmol/L)	< 20.5	10.2
Alkaline phosphatase (Unit/L)	40.0–150.0	97
Aspartate aminotransferase (Unit/L)	8.0–48	10.0
Alanine aminotransferase (Unit/L)	16–63	16
Antinuclear antibodies—ELISA	Negative	Negative
Anti-dsDNA antibodies (IU/mL)	0–200	70.6
C3 (g/L)	0.79–1.52	1.22
C4 (g/L)	0.16–0.38	0.35
Urine protein creatinine ratio (mg/mmol)	< 15	32
24 h urine protein (mg/day)	< 150.00	359.6
Urine examination: Bland with no RBCs or WBCs or casts, +1 protein		
Ultrasound kidneys: Both kidneys show normal size and increased echogenicity. Good corticomedullary differentiation. No obvious stones or focal lesion.		

**Table 2:** Index case investigations. Genetic test results of the index case.

Gene (Isoform)	Phenotype MIM number (mode of inheritance)	Variant	Zygosity	MAF gnomAD (%)	Classification
*IQCB1* (NM_001023570.4)	609254 (AR)	c.1457C > A p.(Ala486Asp) chr3:121491514	Homozygous	0	Uncertain significance

Abbreviations: AR, Autosomal Recessive; gnomAD, Genome Aggregation Database; MAF, Minor Allele Frequency; MIM, Mendelian Inheritance in Man.

## Data Availability

Data that support the findings of this study are available from the corresponding author upon reasonable request.
